# Anti-ARHGAP26 Autoantibodies Are Associated With Isolated Cognitive Impairment

**DOI:** 10.3389/fneur.2018.00656

**Published:** 2018-08-10

**Authors:** Frederik Bartels, Harald Prüss, Carsten Finke

**Affiliations:** ^1^Department of Neurology, Charité – Universitätsmedizin Berlin, Berlin, Germany; ^2^Berlin School of Mind and Brain, Humboldt-Universität zu Berlin, Berlin, Germany; ^3^German Center for Neurodegenerative Diseases (DZNE) Berlin, Berlin, Germany

**Keywords:** ARHGAP26, GRAF1, anti-Ca, medusa-head antibodies, neuronal autoantibodies, cognitive impairment

## Abstract

Autoantibodies against the RhoGTPase-activating protein 26 (ARHGAP26) were originally identified in the context of subacute autoimmune cerebellar ataxia. Further studies identified a wider clinical spectrum including psychotic, affective, and cognitive symptoms. Only a few patients reported so far had evidence of a tumor association. A prospective analysis between January 2015 and December 2017 at the Dept. of Neurology at Charité—Universitätsmedizin Berlin identified 14 patients with ARHGAP26 autoantibodies on a cell-based assay, of which three patients had additional brain immunohistochemistry staining of cerebellar molecular layer and Purkinje cells, who were therefore considered antibody-positive. In all three patients, ARHGAP26 autoantibodies were associated with tumors. In two patients, an isolated cognitive impairment without additional neurological deficits was observed. These cases thus further extend the clinical spectrum associated with ARHGAP26 autoantibodies and strengthen a potential paraneoplastic context.

## Introduction

Autoantibodies against the RhoGTPase-activating protein 26 (ARHGAP26) were originally identified in patients with subacute autoimmune cerebellar ataxia (ACA). The first patient was described in 2010 and presented with limb and gait ataxia, dysarthria and diplopia developing over a period of 2 weeks, followed by hyperekplexia, depression, restlessness, and anxiety. Further tests revealed normal CSF cell count, but intrathecal IgG synthesis and subsequent cerebellar atrophy on MRI. No tumor was detected (1). Two more patients with ARHGAP26 autoantibodies and cerebellar ataxia were reported 3 years later (2). In one of these patients, antibody detection lead to the discovery of ovarian cancer, suggesting ARHGAP26 autoantibodies as a potential marker of a paraneoplastic neurological syndrome (PNS). The other reported patient had weight loss, but no further tumor workup was performed. In an additional case, ARHGAP26 antibodies were not only associated with cerebellar ataxia, but also with cognitive and affective symptoms, indicating a broader clinical spectrum (3). This notion was further supported by the report of a fifth case with recurrent psychotic episodes, but no signs of cerebellar ataxia (4). Recently, two more cases have been described including one with cerebellar ataxia and a history of both breast cancer and melanoma and a second case with cerebellar ataxia, tremor, myoclonus, depression, and mild cognitive deficits (5).

In summary, ARHGAP26 autoantibodies were primarily reported in patients with cerebellar ataxia, but have also been associated with additional clinical features such as psychotic symptoms, depression, and cognitive decline. A tumor was detected in some of these patients, suggesting a potential paraneoplastic etiology.

Here, we report three new cases with predominant cognitive impairment and associated malignancy, further extending the clinical spectrum associated with ARHGAP26 autoantibodies and strengthening their potential paraneoplastic context.

## Methods

### Patients

Patients with a suspected autoimmune-mediated brain disorder seen at the Department of Neurology at Charité—Universitätsmedizin Berlin and 1,055 additional tumor patients were prospectively screened for neuronal autoantibodies between January 2015 and December 2017. Tumor patients had a confirmed diagnosis of melanoma, prostate, lung, breast, gastric/esophageal, or colon cancers, leukemia, or lymphoma and were recruited at the corresponding departments. Patient charts were retrospectively reviewed. The study was approved by the ethics committee of Charité—Universitätsmedizin Berlin and all patients gave written informed consent for publication.

### Antibody detection

ARHGAP26 autoantibodies were detected by cell-based assay (CBA) and immunohistochemistry. Patients were only considered antibody-positive if both assays were positive. The CBA used fixed human recombinant HEK293-cells expressing ARHGAP26 (Euroimmun AG, Lübeck, Germany). For immunohistochemistry, cryosections of brain tissue (rat hippocampus, rat cerebellum, monkey cerebellum, Euroimmun AG) were incubated with patient serum/CSF using indirect immunofluorescence. The previously described IgG cerebellar staining pattern of the molecular layer and Purkinje cells (PC) was considered positive on immunohistochemistry (1).

CBA revealed 14 ARHGAP26-positive patients with serum titers between 1:10 and 1:10,000. Of those, samples from three patients showed typical cerebellar staining on immunohistochemistry and were therefore considered antibody-positive in this study.

### Neuropsychological assessment and cerebral MRI

Detailed cognitive assessment was performed using neuropsychological tests evaluating working memory, verbal, and visuospatial long-term memory, attention, language, executive functions, and premorbid intelligence level. In case 2, the Montreal Cognitive Assessment (MOCA) was performed, including tests for short-term memory, visuospatial abilities, executive function, attention, and language (6). In case 1, MRI data was acquired on a 1.5T Symphony Vision scanner (Siemens, Erlangen, Germany) using a coronal T2w TIRM sequence and a contrast-enhanced MPGRAGE sequence.

## Results

### Case 1

**Figure 1:**
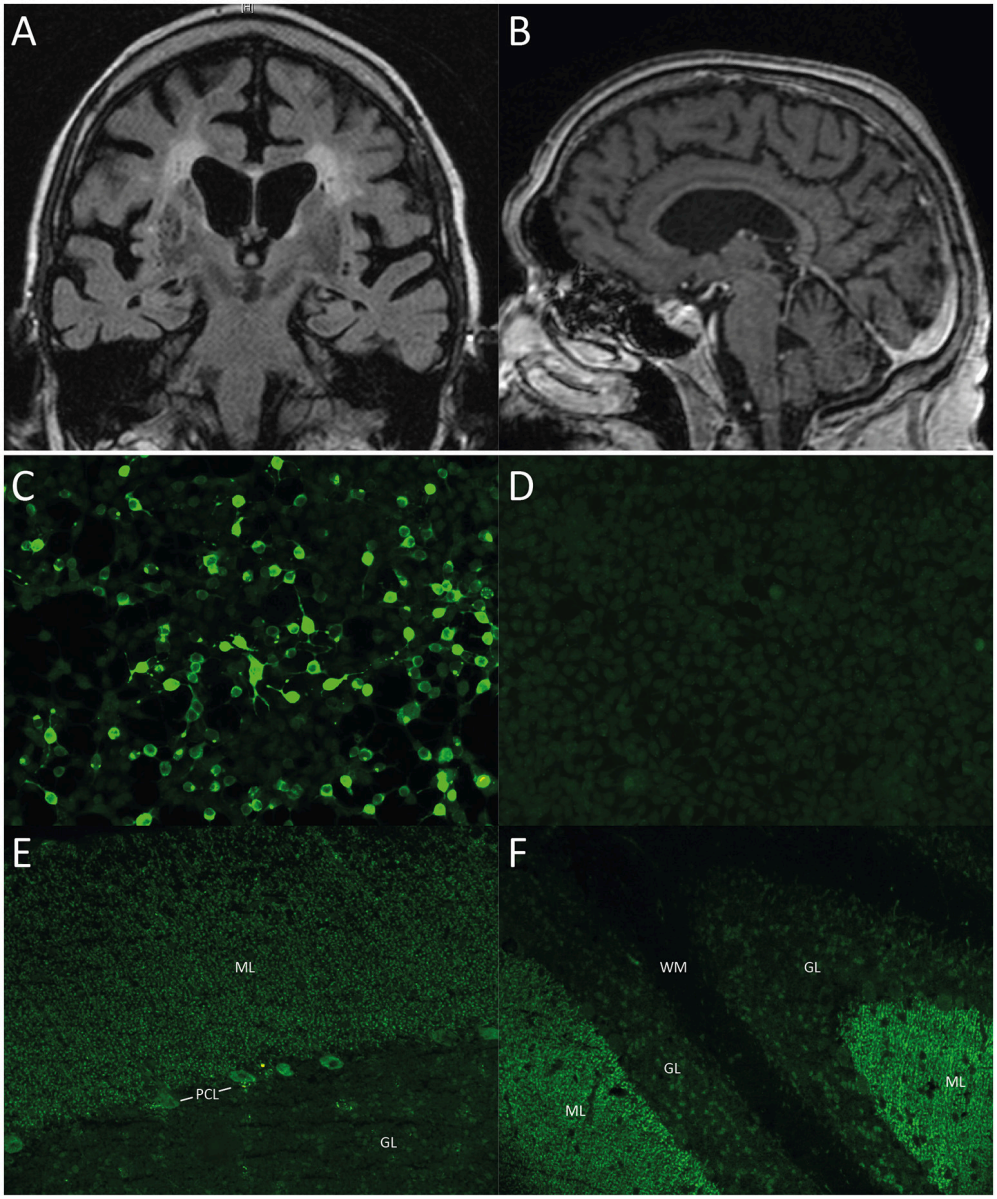
**(A, B)** Cerebral MRI of case 1. **(A)** Coronal T2 TIRM image and **(B)** Contrast-enhanced MPGRAGE image showing whole brain atrophy apparent in the frontal and parietal cortex as well as in the insular region with proportional atrophy of the hippocampus and the cerebellum as well as signs of microangiopathy. **(C–F)** Immunohistochemistry, representative images of case 2. All images taken at 200x magnification. **(C)** Cell-based assay with recombinant ARHGAP26-expressing HEK293-cells, 1:100 dilution, showing binding of patient serum IgG to ARHGAP26-expressing cells. **(D)** Empty-vector control cells after incubation with patient serum (1:100 dilution) demonstrates no binding of patient IgG. **(E)** Cerebellum monkey, 1:100 dilution with patient serum IgG, showing staining pattern of molecular layer (ML) and Purkinje cell layer (PCL). **(F)** Cerebellum rat, 1:100 dilution, Note patient serum IgG binding to molecular layer, Purkinje cell layer (PCL), but not white matter (WM). ML, molecular layer; PCL, Purkinje cell layer; GL, granular layer; WM, white matter.

This 84 year-old patient presented with progressive anterograde amnesia developing within a few weeks, followed by Broca's aphasia, loss of appetite, weight loss, intermittent hyponatremia, gait ataxia, and emotional instability. Neurological examination showed vertical and horizontal saccadic eye movements with occasional ocular flutter, generalized muscular atrophy, brisk tendon reflexes, gait ataxia, marked dysdiadochokinesia, and impaired fine-motor skills. Detailed neuropsychological testing revealed mild cognitive impairment with deficits of attention, word fluency, working, and anterograde verbal memory. Cerebral MRI showed marked generalized atrophy and signs of microangiopathy (Figures 1A,B). Basic CSF studies were unremarkable, protein 14-3-3 was negative. The patient had a medical history of monoclonal gammopathy (MGUS) (IgM-lambda) and multiple cardiovascular risk factors including arterial hypertension (AHT), coronary artery disease (CAD) and minor posterior circulation strokes without persistent neurological deficits. Serological testing revealed ARHGAP26 autoantibodies with a 1:1,000 titer and a typical staining pattern on brain tissue, i.e., IgG staining of cerebellar molecular layer and PCs.

The patient was started on oral methylprednisone 500 mg/d for 3 days, followed by 40 mg/d for 4 weeks and subsequent weekly reduction by 10 mg. In light of a possible PNS, further diagnostic workup was recommended. Follow-up studies revealed a decrease in serum titer to 1:32 with no antibodies detected in CSF. Again, CSF basic studies were unremarkable, but CSF-specific oligoclonal bands (OCBs) were positive. Phospho-TAU/TAU, beta-amyloid and beta-amyloid ratio were unremarkable. Cerebral MRI remained unchanged and a whole-body PET-CT revealed no tumor signs. The patient received four cycles of plasmapheresis with mild improvements of short-term memory and was discharged for rehabilitation.

Three months later, the patient presented with agitation and depression. At this point, serum testing showed an ARHGAP26 antibody titer of 1:100 and the previously documented MGUS (IgM-lambda). Further hematologic work-up revealed a B-cell lymphoma, for which the patient was started on obinutuzumab and chlorambucil.

### Case 2

This 73-year-old patient with prostate cancer presented with slowly progressive memory decline over the last years, mainly having trouble remembering new names and appointments. He had been diagnosed with prostate cancer 10 years before and hepatic metastases were detected a few months prior to presentation. He had a history of asthma and migraine, but had been without symptoms for over 20 years. At the time of presentation, his prostate cancer was treated with docetaxel.

His neurological examination was unremarkable, except for mild tandem gait imbalance. The Montreal Cognitive Assessment (MOCA) revealed mild cognitive impairment with 22/30 points (normal ≥26) with deficits in language, abstraction, verbal memory, and orientation. ARHGAP26 antibodies were detected in serum with a CBA (titer 1:3,200) (Figures 1C,D). Immunohistochemistry identified the typical cerebellar staining of the molecular layer and PCs (dilution 1:1,000) (Figures 1E,F). Interestingly, rat hippocampal staining showed a fine granular-to-smooth pattern (1:320). 6 month later, immunohistochemistry remained highly indicative of ARHGAP26 (1:3,200), while the CBA titer increased to 1:10,000. The patient received no immunosuppressive therapy and died a few months later of metastasized prostate cancer.

### Case 3

This 77-year-old man with gastric adenocarcinoma und lung metastases showed cognitive impairment in a detailed neuropsychological work-up. He was diagnosed with gastric carcinoma following abdominal pain 2 years prior to presentation. The patient, a smoker with 30 pack-years, had a history of CAD, AHT, peripheral artery disease, and chronic obstructive pulmonary disease. Staging revealed a pulmonary nodule that was consistent with a distant metastasis of the gastric adenocarcinoma on biopsy. The patient was started on chemotherapy with four cycles of FLOT regimen (fluorouracil, leucovorin, oxaliplatin, and docetaxel), followed by gastric resection and radiotherapy of the lung metastasis with additional four cycles of adjuvant FLOT chemotherapy.

At the time of presentation, there was no evidence of local carcinoma recurrence. The pulmonary nodule remained stable. Neurological examination was unremarkable. Cognitive testing showed deficits in short-term memory, attention, and executive function. Serum testing revealed autoantibodies against ARHGAP26 on CBA (1:100) and immunohistochemistry (1:100).

Table 1 summarizes clinical and diagnostic features of all previously reported ARHGAP26-positive patients including the cases above.

**Table 1:** Clinical and diagnostic characteristics of all reported ARHGAP26-positive patients.

	**Age**	**Gender**	**Symptoms**					**Cerebral MRI**	**CSF studies**	**ARHGAP26 antibody**		**Tumor**
			**Cerebellar**	**Psychotic**	**Affective**	**Cognitive**	**Other**			**Titer serum**	**Titer CSF**	
Patient 1^a^	33	Female	Limb and gait ataxia, nystagmus, dysarthria	–	Depression		Hyperekplexia	Cerrebellar atrophy	Pleocytosis(44/μl). BBB disruption, OCBs pos	1:6,000	1:2,000	–
Patient 2^b^	68	Female	Gait ataxia, dysarthria, nystagmus, dizziness, nausea/vomiting, cerebellar ataxia	–	–	–	–	Empty sella, Cerrebellar atrophy	–	1:32,000	–	Ovarian cancer
Patient 3^b^	38	Male	Dysarthria, gait ataxia, dysarthria, nystagmus, dizziness				Weight loss	Cerrebellar atrophy	5 cells/ul: OCBs pos	1:3,200	–	–
Patient 4^c^	24	Male	Ataxia, dysarthria, nystagmus, oscillopsia		Flattended affect	Cognitive impairment (deficits in attention, executive function, working memory, verbal learning and recall, and spatial recognition)	Weight loss headache	Cerrebellar atrophy	BBB disruption, OCBs pos	1:20,000	1:240	–
Patient 5^d^	34	Female	–	Reccurent psychotic symptoms (impressive and aggressive behaviors, altered personality, socially inappropriate actions, mutism, apathy)	Suicidal thoughts	–	Headache	Normal	Normal	1:1,000	pos.	–
Patient 6^e^	57	Female	Limb and gait ataxia, saccadic eye movement, dizziness	–	–	–	Cutaneous hematoma	Normal	Normal	1:32	–	History of breast cancer, melanoma
Patient 7^e^	37	Female	Limb and gait ataxia, nystagmus, dysarthria	–	Depression	–	–	Normal	BBB disruption, OCBs pos	1:100	–	–
Case 1 (Patient 8)	84	Male	Limb and gait ataxia, saccadic eye movement, ocular flutter	–	Emotional instability	Cognitive impairment (deficits in attention, working memory, semantic word fluency, and anterograde verbal memory) Cognitive impairment (memory deficit)	Hyperekplexia myoclonic jerks. Loss of appetite, weight loss, intermittent hyponatremia	Genralized atrophyl	OCBs pos	1:1,000	–	B-cell lymhoma
Case 2 (Patient 9)	73	Male	–	–	–	–	–	–	–	1:10,000	–	Prostrate cancer
Case 3 (Patient 10)	77	Male	–	–	–	Cognitive impairment (deficits in short-term memory, attention and executive function)	–	–	–	1:100	–	Gastric adenocarcinoma

a *(1)*;

b *(2)*;

c *(3)*;

d *(4)*;

e *(5)*.

## Discussion

We here describe three new ARHGAP26-positive cases with isolated cognitive impairment in two patients and a tumor association in all three cases, suggesting a broader clinical spectrum and highlighting the importance to screen antibody-positive patients for malignancies.

The previously described clinical spectrum associated with ARHGAP26 autoantibodies includes cerebellar ataxia, but also psychotic, affective and cognitive symptoms (1–5). In line with the initial context of cerebellar ataxia, immunohistochemistry revealed binding to cerebellar molecular layer and PC cytoplasm and membrane (1). While a pathogenic role of ARHGAP26 autoantibodies remains unknown, tissue staining would be consistent with a clinical effect of pure cerebellar syndrome. However, other associated clinical symptoms such as depression, psychotic behavior and cognitive deficits would be more difficult to explain. Here, we observed predominant cognitive impairment in three ARHGAP26-positive patients - with in fact isolated cognitive deficits without other neurological symptoms in two of the patients. Affected cognitive domains included attention, short-term and working memory, verbal memory, semantic word fluency, and executive function.

One possible explanation for cognitive deficits in ARHGAP26-positive patients is that autoantibodies not only bind to cerebellum, but also other brain structures such as limbic regions including the hippocampus. Indeed, ARHGAP26 was found to be expressed in a subset of hippocampal neurons (4, 7). In a previous ARHGAP26-positive case with isolated psychiatric symptoms the authors concluded that the lack of cerebellar symptoms in that patient suggests other brain regions to be involved (4). Furthermore, they pointed out that in other antibody-associated neurological disorders, a wide clinical spectrum of one single antibody (e.g., anti-Hu, anti-AQ4) is common, presumably due to widespread expression of the antigen. Interestingly, in our case 2, immunohistochemistry revealed hippocampal staining, suggesting a potential autoimmune response to the limbic system, although the presence of another, yet undefined antibody cannot be excluded.

Alternatively, cognitive symptoms could directly be mediated by cerebellar dysfunction, as conceptualized by the cerebellar cognitive–affective syndrome (Schmahmann's syndrome) (8–10). Schmahmann's syndrome occurs in patients with isolated cerebellar disease and includes deficits of attention, working and verbal memory, visuo-spatial cognition, executive function, language as well as behavior and affect (11). It is therefore well-suited to explain the cognitive and affective symptoms in patients with ARHGAP26 antibodies. However, isolated cognitive deficits in Schmahmann's syndrome have not been reported so far. Associated anatomical regions include large bilateral or pancerebellar damage (8). Due to the lack of post-mortem studies in ARHGAP26-positive patients, the targeted cerebellar regions are unknown, but MRI studies revealed generalized cerebellar atrophy. Therefore, it seems plausible that isolated cognitive dysfunction could be part of a cerebellar cognitive-affective syndrome in ARHGAP26-positive patients (3).

An underlying tumor was only found in two of the seven previously reported cases (2, 5), whereas all three of the here described patients had a cancer diagnosis before or revealed on further work-up. Autoantibodies targeting intracellular antigens are frequently associated with underlying malignancy and can precede cancer diagnosis by up to 15 months (12, 13). Therefore, delayed tumor detection in previous cases cannot be excluded. Indeed, repeated tumor screening in case 1 led to the diagnosis of B-cell lymphoma 12 months after initial presentation. With these three new cases, now 50% of all reported ARHGAP26-positive patients had a tumor-association, emphasizing the importance to screen for underlying malignancy. Interestingly, ARHGAP26 was found to be expressed in most samples of prostate cancer, suggesting a possible trigger in case 2 (7).

Immunosuppressive therapy was only administered in one patient. Here, mild improvement of short-term memory was observed after steroids and plasmapheresis, even before tumor detection and treatment. This suggests a potential benefit of immunosuppressive therapy. Tumor treatment was initiated or continued in all patients. While one patient died of his prostate cancer, long-term outcome of the other patients remains to be seen. Previous cases reported little effect of immunosuppressive therapy at best, ideally stabilizing patients with the existing deficits (5). Long-term clinical follow-up and evaluation of patient outcome after immunosuppressive and tumor therapy should be addressed in future studies.

We considered patients antibody-positive only when being positive in both assays, CBA and immunohistochemistry on brain sections. Of the 11 patients that were positive on CBA only (i.e., without the corresponding immunohistochemistry pattern), three underwent neuropsychological assessment. Interestingly, two of these patients also had isolated cognitive impairment. It is unclear whether in these cases the CBA is more sensitive than immunohistochemistry, similar to assays with other antibodies such as against the NMDA receptor. Alternatively, antibodies may bind to an epitope that is present only on recombinantly expressed antigens in the CBA or which is lost on brain sections, e.g., due to tissue processing including fixation. Further clinical correlations with more patients are required to disentangle the significance of isolated positive CBAs.

In summary, we here describe three new cases with ARHGAP26 autoantibodies with tumor association that presented with predominant cognitive impairment. This allows for the following conclusions for clinical practice: (1) The spectrum of ARHGAP26-associated symptoms is broader than initially expected and also includes isolated cognitive impairment; (2) A positive ARHGAP26 antibody-test should prompt the search for an underlying malignancy; and (3) ARHGAP26-mediated autoimmune encephalopathy is a potential, yet rare differential diagnosis in patients with cognitive impairment.

## Author contributions

Data was collected by FB, HP, and CF. Figures and tables were created by FB with support of HP and CF. Manuscript was written and edited by FB, HP, and CF.

### Conflict of interest statement

The authors declare that the research was conducted in the absence of any commercial or financial relationships that could be construed as a potential conflict of interest.
